# Number of dental abutments influencing the biomechanical behavior of tooth‒implant-supported fixed partial dentures: A finite element analysis

**DOI:** 10.34172/joddd.2020.047

**Published:** 2020-12-07

**Authors:** Janaina Cordeiro de Oliveira, Mariane Beatriz Sordi, Ariadne Cristiane Cabral da Cruz, Raquel Virgínia Zanetti, Ederson Aureo Golçalves Betiol, Sérgio Roberto Vieira, Artêmio Luiz Zanetti

**Affiliations:** ^1^Department of Dentistry, Federal University of Santa Catarina, Florianópolis, Brazil; ^2^Postgraduate in Dentistry, Federal University of Santa Catarina, Florianópolis, Brazil; ^3^Laboratory of Applied Virology, Federal University of Santa Catarina, Florianópolis, Brazil; ^4^Postraduate in Dentistry, São Leopoldo Mandic University, Campinas, Brazil; ^5^Department of Dentistry, Federal University of Paraná, Curitiba, Brazil; ^6^Private Practice, Curitiba, Brazil; ^7^Department of Prosthodontics, Faculty of Dentistry, University of São Paulo, São Paulo, Brazil

**Keywords:** Dental prosthesis, Finite element analysis, Fixed partial denture, Implant-supported

## Abstract

**Background.** Local or systemic issues might prevent installing a sufficient number of dental implants for fixed prosthetic rehabilitation. Splinting dental implants and natural teeth in fixed dentures could overcome such limitations. Therefore, this study aimed to evaluate the influence of the number of dental abutments in the biomechanics of tooth‒implant-supported fixed partial dentures (FPDs). The null hypothesis was that increasing the number of abutment teeth would not decrease the stress over the abutments and surrounding bone.

**Methods.** Left mandibular lateral incisor, canine, premolars, and molars were reconstructed through computed tomography and edited using image processing software to represent a cemented fixed metal‒ceramic partial denture. Three models were set to reduce the number of abutment teeth: 1) lateral incisor, canine, and first premolar; 2) canine and first premolar; 3) the first premolar. The second premolar and first molar were set as pontics, and the second molar was set as an implant abutment in all the models. Finite element analyses were performed under physiologic masticatory forces with axial and oblique loading vectors.

**Results.** After simulation of axial loads, the stress peaks on the bone around the implant, the bone around the first premolar, and prosthetic structures did not exhibit significant changes when the number of abutment teeth decreased. However, under oblique loads, decreasing the number of abutment teeth increased stress peaks on the surrounding bone and denture.

**Conclusion.** Increasing the number of dental abutments in tooth‒implant-supported cemented FPD models decreased stresses on its constituents, favoring the prosthetic biomechanics.

## Introduction

Osseointegrated implants have become a highly predictable treatment solution in prosthetic rehabilitation of fully or partially edentulous patients. There are retrospective studies in the literature, reporting a cumulative survival rate of implants up to 87.8% after 36 years of follow-up.^1-4^ Despite the great success of the original protocol for dental implant rehabilitation proposed by Brånemark, implant designs, surgical techniques, and surface/material modifications have undergone constant changes over time.^5-7^

Several approaches have been reported to enable dental rehabilitation. Moreover, with the extensive use of dental implants worldwide,^8^ variations in treatment plans are even more diverse. In some situations, clinicians might face complex cases where it is not feasible to install a sufficient number of implants to enable implant-supported rehabilitation due to local or systemic diseases. In these cases, splinting dental implants and natural teeth might be one alternative for fixed partial dentures (FPDs).^9,10^ This modality of prosthetic rehabilitation could increase patients’ acceptance and reduce treatment costs and complexity.^11^ Additionally, splinting teeth and implants for FPD has an estimated 5-year survival rate of 94.73% and a 10-year survival rate of 77.77%.^12^

Nevertheless, several limitations and controversial results have been related to tooth‒implant-supported FDPs.^9,10^ The main peculiarity of these dentures is that their behavior under occlusal loads applied to implants is different from teeth since the periodontal ligament is capable of absorbing tensions and minor dental movements, which does not occur with osseointegrated implants.^13-15^ Consequently, tooth‒implant-supported FPD is not an optimal approach. However, this treatment option is recommended when the clinician faces limitations regarding the anatomical structures, patient’s compromised systemic condition, proprioception, financial issues, and/or patient preferences. Also, the extraction of healthy teeth to avoid tooth‒implant connections should be avoided.^11,16-21^ Therefore, the evaluation and quantification of tensions over the supporting tissues and abutment systems in tooth‒implant-supported FPDs are essential since damage to prosthetic components or biological structures is attributed to biomechanics.^11,14^

The finite element analysis (FEA) is an important tool for the simulation and analysis of tensions, displacements, and deformations in implants and prosthetic abutments, and evaluating the integrity at the bone level. The FEA allows an analysis of relevant parameters by developing a mathematical model and virtual application of load in different directions and magnitudes on a model that represents a structure very close to the reality under study.^11,22,23^

Thus, considering the possibility of splinting dental implants and natural teeth through an FPD in the rehabilitation of partially edentulous patients, studies demonstrating the effect of the number of dental abutments on the biomechanics of these dentures should be useful.^11^ Therefore, this study aimed to evaluate the influence of the number of dental abutments on the biomechanical behavior of tooth‒implant-supported FPDs by FEA of the prosthesis structures, the supporting bone, and the abutments (tooth and implant). The null hypothesis was that increasing the number of abutment teeth would not decrease the stress over abutments and the surrounding bone.

## Methods

### Virtual reconstruction of computed tomography (CT) and model virtual edition

**Figure 1:**
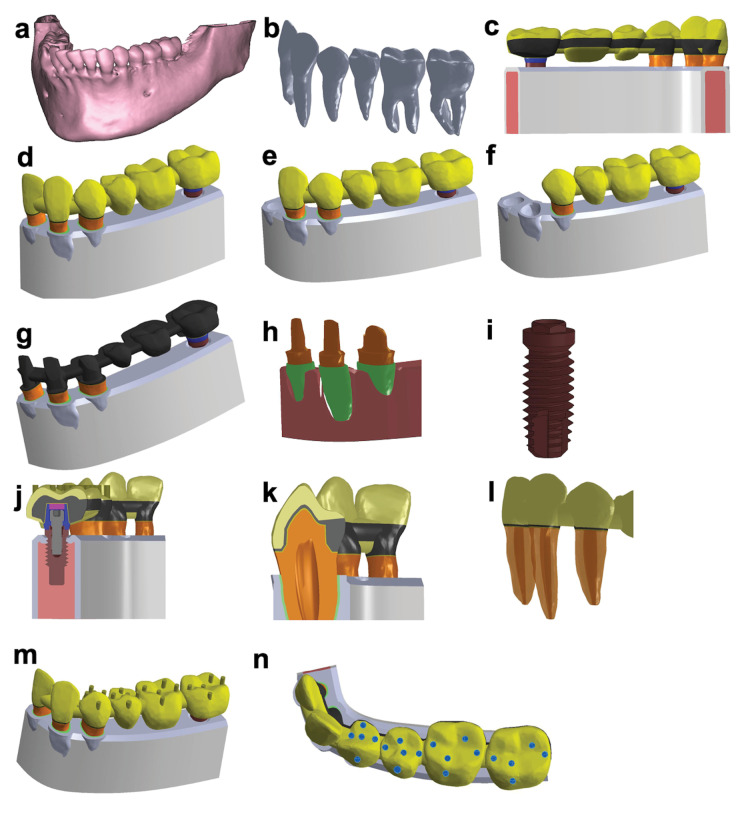
Model editing. a) Digital reconstruction of the mandible in a three-dimensional model obtained from computed tomography. b) The positions corresponding to the left mandibular lateral incisor, canine, first premolar, second premolar, first molar, and second molar were extracted from the digital model. c) Lingual and d) vestibular views of the virtual tooth‒implant-supported FPD. In yellow, the porcelain; in dark gray, the infrastructure; in orange, the dentin; in brown, the implant; in gray, the cortical bone; and in blue, the abutment. d) Model 1; e) Model 2; f) Model 3. g) Model 1 without porcelain, demonstrating the metal FPD infrastructure. h) Abutment tooth preparation in orange, periodontal ligament in green, and bone marrow in pink. i) External hex cylindrical titanium implant (10 mm in height and 4.1 mm in platform diameter). j) Screw-retained custom titanium implant abutment measuring 4.1 mm in platform diameter. Cross-section of implant in a buccolingual direction showing the abutment, implant, and screw interfaces. k) Cross-section of the left mandibular first premolar in a buccolingual direction, showing dentin (orange), cement line (dark yellow), cortical bone (gray), periodontal ligament (green), infrastructure (dark gray), and porcelain (light yellow). l) Abutment teeth. m) Cylinder positions simulating occlusal contacts (crushing, smooth strands, and opposing pits). n) Occlusal view of the cylinders simulating occlusal contacts, which were arranged to simulate a stable occlusion.

After approval by the Human Ethics Committee, following the Helsinki Declaration, a written consent form was signed so that the patient underwent a volumetric computed tomography (CT, i-CAT, Xoran Technologies, Ann Arbor, USA) scan to obtain the digital model. The CT scan was performed to analyze the mandibular region in transverse sections of 0.25 mm with 212 cuts. The cross-sections were recorded in DICOM (digital imaging and communications in medicine standard) and employed to reconstruct the mandible in a three-dimensional (3D) model (Figure 1a). From this model, the positions corresponding to the left mandibular lateral incisor, canine, first and second premolars, and first and second molars were extracted (Figure 1b).

The 3D model was then exported to Ansys Design Modeler v10 software (Ansys Inc., Canonsburg, PA, USA) for virtual edition. An FPD composed of the left mandibular lateral incisor, canine, and first premolar as abutment teeth, second premolar and first molar as pontics, and second molar as implant abutment was constructed. Lingual and vestibular views of the virtual FPD are represented in Figures 1c and 1d, respectively.

### Virtual study models

The three different models aimed to evaluate the impact of loads in terms of the number of dental abutments on the FPD with tooth‒implant splinting. The models consisted of cemented FPD divided as follow: Model 1: lateral incisor, canine, and first premolar as abutment teeth (Figure 1d); Model 2: canine and first premolar as abutment teeth (Figure 1e); and Model 3: first premolar as abutment tooth (Figure 1f).^9^ Second premolar and first molar teeth were set as pontics, and the second molar was set as implant abutment for all models. The models were set with the following characteristics:

### Fixed partial denture

The FPD infrastructure consisted of chromium‒cobalt alloy with a minimum thickness of 0.3 mm, and an inclined plane ridge under the pontic was selected to favor prosthesis hygiene and esthetics (Figure 1g). The dental coronary morphology was designed using feldspar porcelain with a minimum thickness of 0.9 mm covering the metallic infrastructure.

### Abutment teeth

The abutment teeth were prepared with a thickness of 1.2 mm at the cervical end and 1.5 mm at the occlusal surface. Expulsive preparation was about 6 degrees, with a beveled shoulder finish line (Figure 1h). The periodontal ligament around the roots was approximately 0.25-mm thick, and cancellous bone was set under the alveolar bone.

### Implants and implant abutments

External hex cylindrical titanium dental implants (Nobel Biocare, Kloten, Switzerland) with a height of 10 mm and a platform diameter of 4.1 mm (Figure 1i) were employed. The custom dental implant titanium abutment with 4.1 mm of platform diameter was screw-retained (Figure 1j).

### Other prosthetic/anatomical structures

A zinc phosphate cement line of about 0.1 mm thickness was set between teeth/implant abutments and prosthesis (Figure 1k). The cortical bone around the periodontal ligament was approximately 0.7 mm in thickness. Lekholm and Zarb^24^ classified bone density into four types. To represent this study model, bone type III was selected since it represents the average density of maxillary bones. The pulp tissue was not considered in this simulation to decrease the computational load (Figure 1l). The enamel cylinders distributed on occlusal/incisal surfaces of the prosthesis were used to simulate tooth contacts and standardize contact points. The cylinders were arranged to simulate a normal occlusion (Figures 1m and 1n).

### Simulation

All models were exported from Ansys Design Modeler to the Ansys Workbench v10 finite element simulation software (Ansys Inc., Canonsburg, PA, USA). To obtain the results, the program required several data, such as Young’s modulus (elasticity) and Poisson’s coefficient (deformation) of the different structures described above. Thus, each element of the models was configured with Young’s moduli and Poisson’s coefficients from the classical literature,^13,14,25-27^ as shown in Table 1.

**Table 1:** Mechanical properties of the studied materials

**Material**	**Young’s Modulus (MPa)**	**Poisson’s Coefficient**
Dentin	18600.0	0.31
Periodontal ligament	68.9	0.45
Cortical bone	13700.0	0.30
Cancellous bone	1370.0	0.30
Feldspar porcelain	69000.0	0.30
Commercially pure titanium	110000.0	0.35
Enamel	84100.0	0.20
Zinc phosphate cement	22400.0	0.25
Chrome-cobalt alloy	218000.0	0.33

The contacting surfaces were considered perfect unions, except for the contacts between the implant abutment and zinc phosphate cement and between the dentin and zinc phosphate cement, configured with a friction coefficient of 0.2 due to the mechanical cement imbrication that results in a non-perfect adhesion between these structures.^28^ Rigid supports were added on the lower and lateral sides of bone margins to simulate the mandible. Model 1 was generated first, and the other models were configured with the suppression of the lateral incisor (Model 2), and lateral incisor and canine (Model 3). Loads were applied in each model in two configurations: (1) axial loads parallel to the long axis of the tooth and (2) oblique loads with a 45º to the long axis of the tooth. Loads of 30 N were used for premolars and 50 N for molars.^29,30^

The mesh was generated with tetrahedral elements, which produce smaller deviations (deformations), resulting in meshes with 1 920 736 nodes and 1 125 143 elements. All the models were elucidated (Windows XP X64, Intel Core 2 Quad Q6600, 8 Gb RAM), and graphical and numerical data plots were recorded, evaluated, and compared.

## Results

**Figure 2:**
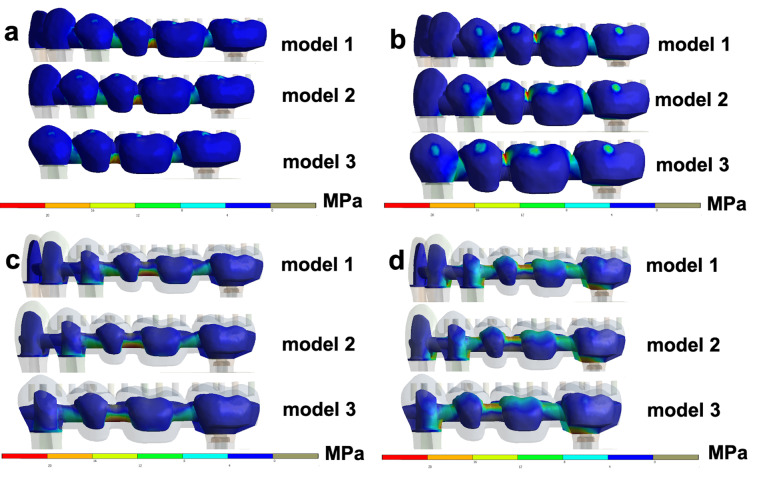
Stress peaks on the porcelain and infrastructure of the FPD under axial loads. a) Traction stresses on the porcelain; b) Compression stresses on the porcelain; c) Traction stresses on the infrastructure; d) Compression stresses on the infrastructure.

**Figure 3:**
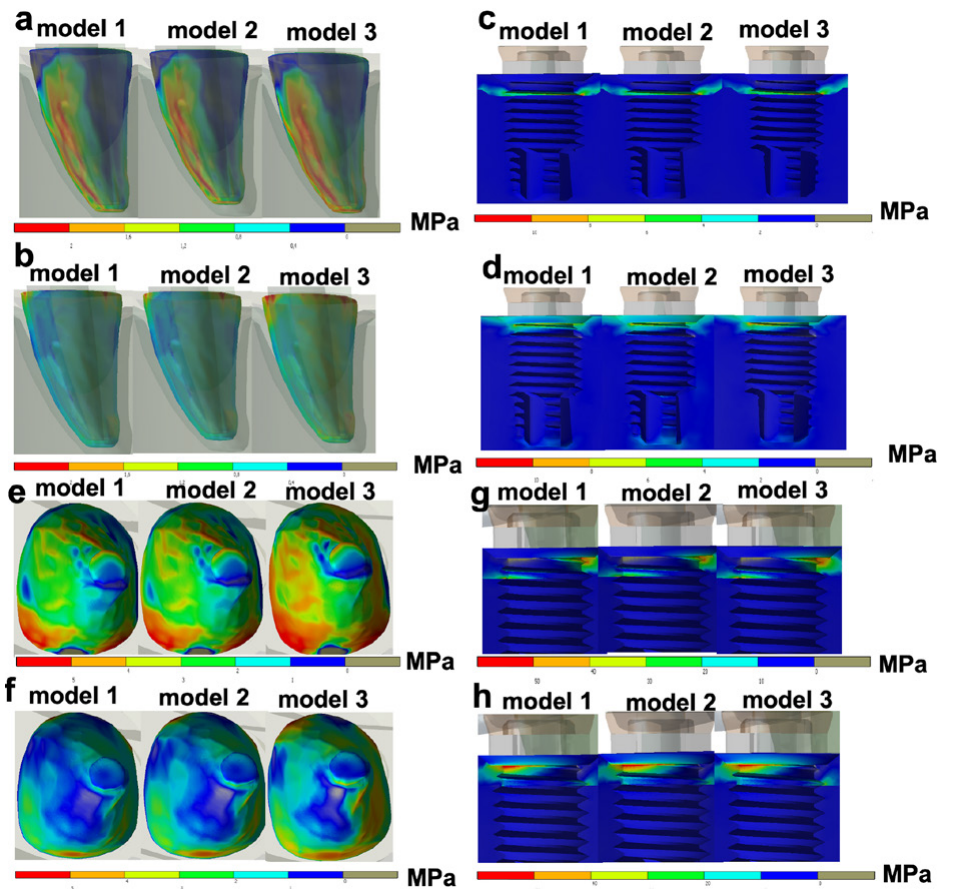
Stress peaks on the bone around abutments (tooth and implant) under axial or oblique loads. a) Traction stresses on bone around the first premolar under axial load in a vestibular view with sectioned models; b) Compression stresses on the bone around the first premolar under axial load in a vestibular view with sectioned models; c) Traction stresses on the bone around implant under axial load in a vestibular view; d) Compression stresses on the bone around the implant under axial load in a vestibular view; e) Traction stresses on the bone around the first premolar under oblique load in an occlusal view; f) Compression stresses on the bone around the first premolar under oblique load in an occlusal view; g) Traction stresses on the bone around the implant under oblique load in a distal view; h) Compression stresses on the bone around the implant under oblique load in a distal view.

**Figure 4:**
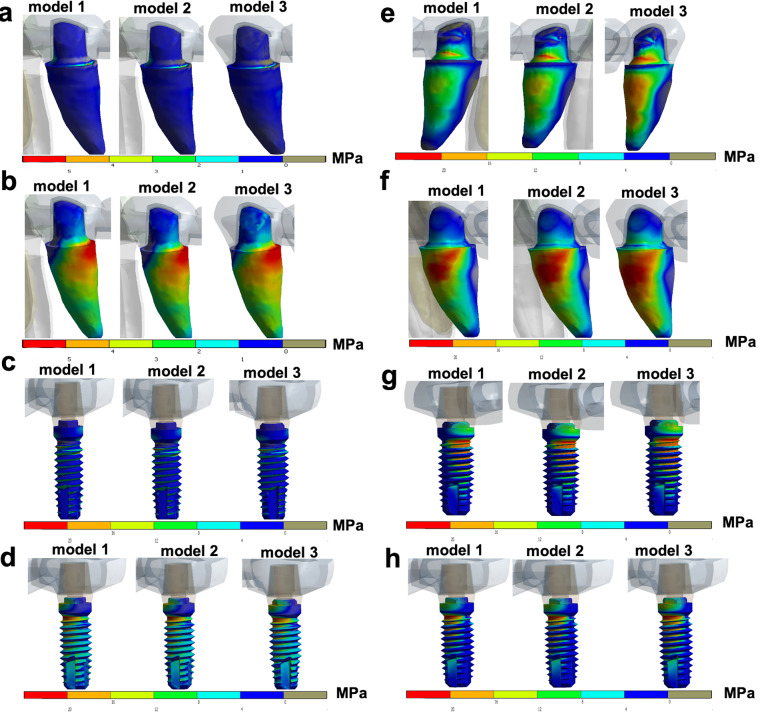
Stress peaks on the abutments (tooth and implant) under axial or oblique loads. a) Traction stresses on the first premolar under axial load; b) Compression stresses on the first premolar under axial load; c) Traction stress on the implant under axial load; d) Compression stresses on the implant under axial load; e) Traction stresses on the first premolar under oblique load; f) Compression stresses on the first premolar under oblique load; g) Traction stress on the implant under oblique load; h) Compression stresses on the implant under oblique load

According to Table 2, when axial loads were applied on the FPD, similar outcomes for stress peaks were obtained in models 1, 2, and 3, with slightly higher values (MPa) for model 2 (Figure 2). Table 3 displays data of tensions after the simulation of axial and oblique loads on the bone around the implant abutment and abutment tooth. The stress peaks on the bone around abutments were higher for model 3 in all the simulated situations except for axial traction loads over implant abutments (Figure 3). Oblique loads revealed that model 3 had the highest stress values for the bone around both implant abutment and abutment tooth, especially for compression (Figure 3). The data in Table 4 presents tensions under axial and oblique loads over implant abutment and abutment tooth. Model 3 revealed the highest stress peak values for all the simulated situations, especially for oblique loads, over both the tooth and implant (Figure 4). Compression forces led to the greatest differences in stress peaks among the models.

**Table 2:** Stress peaks (traction and compression) on the porcelain and infrastructure of the FPD under axial loads (MPa)

**Model**	**Traction Porcelain**	**Compression Porcelain**	**Traction Infrastructure**	**Compression Infrastructure**
1	52.76	68.33	36.33	104.92
2	53.24	68.99	38.06	102.21
3	48.92	62.05	38.02	86.10

**Table 3:** Stress peaks on the bone around abutments (tooth and implant) under axial or oblique loads (MPa)

**Load**	**Model**	**Traction** **Bone around first premolar**	**Compression** **Bone around first premolar**	**Traction** **Bone around implant**	**Compression** **Bone around implant**
Axial	1	4.09	2.47	27.78	34.68
	2	4.14	2.59	27.61	35.33
	3	5.22	3.19	24.44	37.87
Oblique	1	14.62	8.97	120.08	147.68
	2	17.53	10.93	127.35	156.53
	3	26.35	22.07	141.2	170.63

**Table 4:** Stress peaks on the abutments (tooth and implant) under axial or oblique loads (MPa)

**Load**	**Model**	**Traction** **First premolar abutment**	**Compression** **First premolar abutment**	**Traction** **Implant abutment**	**Compression** **Implant abutment**
Axial	1	7.37	13.55	27.65	113.5
	2	7.45	13.85	28.08	115.15
	3	7.13	14.71	28.4	138.19
Oblique	1	26.88	26.97	587.45	574.55
	2	36.99	30.17	637.68	614.09
	3	54.86	40.92	715.71	681.59

## Discussion

In several clinical situations, local or systemic issues prevent installing a sufficient number of implants for implant-supported rehabilitation. In such situations, splinting natural teeth and implants might allow fixed prosthetic rehabilitation.^9,10^ However, these prostheses connect components that are biomechanically distinct since the periodontal ligament around the tooth root is capable of absorbing tensions and minor dental movements, which does not occur with osseointegrated implants.^13-15^ Thus, studies demonstrating the effect of the number of dental abutments on the biomechanics of tooth‒implant-supported FPD would be helpful for clinicians.^11^

Therefore, this study aimed to evaluate the influence of the number of dental abutments on the biomechanical behavior of tooth‒implant-supported FPD by FEA of the prosthesis structures, the supporting bone, and the abutments (tooth and implant). In summary, we observed that after simulation of axial loads, the stress peaks on the bone around the first premolar, the bone around the implant, and the porcelain and prosthesis infrastructure did not exhibit major changes when the number of abutment teeth decreased. However, under oblique loads, decreasing the number of abutment teeth in the analyzed models led to increased stress peaks on the surrounding bone and prosthesis. Thus, regarding tooth‒implant splinting of FPD, the number of abutment teeth seems to decrease the stress peaks on prosthetic components and surrounding bone, favoring the biomechanics of the denture when this type of rehabilitation is required.

Similar studies have been published before. Lin et al^31^ evaluated the effects of loading condition, the number of splinted teeth, and rigid/non-rigid connectors on the mechanical responses of bone, prosthesis, and implant, and the interactions between rigid/con-rigid connectors and the number of splinted teeth in a tooth‒implant-supported FPD through FEA. However, they did not use a computed tomography of a human model and used a 200-N loading force over their models. Later on, Lin et al^32^ investigated the biomechanical interactions in tooth‒implant-supported FPDs with variations in periodontal support, implant system, number of splinted teeth, and load type using FEA. This time, the group used a computed tomography-generated model, which more closely resembled real conditions, and a 200-N loading force was applied.

Additionally, Lanza et al^33^ evaluated a metal‒ceramic fixed tooth‒implant-supported denture through FEA by varying the number of teeth used as abutments. They simulated a model in the posterior region of the maxilla that was not generated from a computed tomography scan of a human model, and 100-N loading forces were applied. Therefore, the novelty of the present study is the fact that a varying number of teeth were used as abutments up to two, using a computed tomography model to better simulate real human clinical conditions.

Splinting natural teeth and implants during the rehabilitation of partial edentulism is not an optimal approach; however, it might be useful to reduce distal cantilevers or avoid removable partial dentures. Thus, whenever suitable and justified, such a treatment option becomes a valid alternative, especially if it reduces treatment complexity, costs, and patient acceptance. Therefore, tooth‒implant-supported FPDs are recommended when the clinician faces limitations related to anatomical structures, patient’s compromised systemic condition, proprioception, financial issues, and/or patient preferences. In addition, the extraction of healthy teeth to avoid tooth‒implant connections should be avoided.^11,16-21^

The influence of the number of splinted teeth has been investigated in previous biomechanical studies, where the authors have claimed that tooth splinting might decrease displacement and stress concentrations, especially in the periodontally compromised dentition.^17,31,34^ To reduce the risk of tooth intrusion, it was suggested a minimum of two natural teeth be connected when tooth‒implant connections are considered. Indeed, connecting teeth in a splint system is one way to decrease mobility in periodontally compromised dentitions for FPDs.^31,35^ Therefore, before splinting natural teeth and implants, it is crucial to consider some factors, such as prosthetic design, occlusion, parafunctional activity, tooth condition, periodontal health, bone quality and quantity, implant inclination, implant size, and patients’ expectations and motivation.^11^

It is essential to observe the differences between axial and oblique loads. Certainly, chewing motion is more complex than the evaluation of a single load axis; however, axial loads are less common during mastication, while oblique loads better represent the cyclic movement.^36,37^ Hence, it is important to remember that oblique forces generate greater tensions in the prosthesis and supporting bone, which might destabilize prosthetic rehabilitation. Mastication load values vary meaningfully according to the literature, which might be attributed to several reasons, including the use of different measurement methods, the patient’s dental structure, orofacial muscle structure, age, and gender, among others.^38^ Herein, we applied loads of 30 N for premolars and 50 N for molars since they are physiological. Nevertheless, pathological masticatory forces might have peaks of 350 N in the posterior region and 200 N in the anterior region.^39,40^

Observing the obtained results, it was evident that the stress peaks reached higher values for implants than teeth in all the simulated models, under both axial and oblique forces, and in both traction and compression. This is due to the different behavior of osseointegrated implants compared to natural teeth. The different mobility patterns of implants and teeth make the biomechanical behavior of the entire system complicated.^41^ An osseointegrated implant can move only 10 µm in the apical direction, whereas teeth with healthy periodontal ligament can move 25‒100 µm.^42^ This movement disparity might cause relative motion of the tooth‒implant superstructure when the splinted system is under occlusal loads. During loading, the bending movement induced by the misfit between the implant and tooth might result in the fracture of the implant or prosthetic components, increased marginal bone loss, or even the loss of osseointegration.^43^

## Conclusion

Since axial and oblique loads might occur during mastication, regardless of the occlusal configuration, the findings of this study indicated that in a tooth‒implant-supported FPD, a higher number of abutment teeth included in the rehabilitation reduces the stress peaks on the prosthesis and supporting bone, favoring the biomechanics of the denture. Nevertheless, this rehabilitation approach should be considered only for cases with limitations, where the installation of an appropriate number of dental implants or tooth extraction must be avoided. Finally, longitudinal clinical studies are recommended for further clarification.

## Authors’ Contributions

JCO performed the acquisition, analysis, and interpretation of data, drafted the manuscript, and revised the manuscript critically. MBS performed the interpretation of the data, drafted the manuscript, and revised the manuscript critically. ACCC interpreted the data, drafted the manuscript, and revised the manuscript critically. RVZ interpreted the data and revised the manuscript critically. EAGB interpreted the data and revised the manuscript critically. SRV interpreted the data and revised the manuscript critically. ALZ interpreted the data and revised the manuscript critically.

## Acknowledgments

Mariane Beatriz Sordi was supported with scholarship by the Coordenação de Aperfeiçoamento de Pessoal de Nível Superior - Brasil (CAPES).

## Funding

This study was conducted with the resources of the authors.

## Competing Interests

The authors declare no competing interests.

## Ethics Approval

The present study was approved by the Human Ethics Committee of the São Leopoldo Mandic University (2009/0001).

## References

[1] Brånemark PI, Adell R, Breine U, Hansson BO, Lindström J, Ohlsson A (1969). Intra-osseous anchorage of dental prostheses I Experimental studies. Scand J Plast Reconstr Surg.

[2] Brånemark PI, Lindström J, Hallén O, Breine U, Jeppson PH, Ohman A (1975). Reconstruction of the defective mandible. Scand J Plast Reconstr Surg.

[3] Brånemark PI, Hansson BO, Adell R, Breine U, Lindström J, Hallén O (1977). Osseointegrated implants in the treatment of the edentulous jaw Experience from a 10-year period. Scand J Plast Reconstr Surg Suppl.

[4] Chrcanovic BR, Kisch J, Albrektsson T, Wennerberg A (2018). A retrospective study on clinical and radiological outcomes of oral implants in patients followed up for a minimum of 20 years. Clin Implant Dent Relat Res.

[5] Dohan Ehrenfest DM, Coelho PG, Kang BS, Sul YT, Albrektsson T (2010). Classification of osseointegrated implant surfaces: materials, chemistry and topography. Trends Biotechnol.

[6] Esposito M, Ardebili Y, Worthington HV (2014). Interventions for replacing missing teeth: different types of dental implants. Cochrane Database Syst Rev.

[7] Junker R, Dimakis A, Thoneick M, Jansen JA (2009). Effects of implant surface coatings and composition on bone integration: a systematic review. Clin Oral Implants Res.

[8] Gaviria L, Salcido JP, Guda T, Ong JL (2014). Current trends in dental implants. J Korean Assoc Oral Maxillofac Surg.

[9] Kumar TA, Chander NG, Jei JB (2018). Tooth implant connected fixed partial denture: 3-year follow-up. SRM J Res Dent Sci.

[10] Singh NS, Singh YS, Singh WR, Mehta P (2012). Natural teeth and implant supported fixed partial denture: a case report. Quintessence Int.

[11] Al-Omiri MK, Al-Masri M, Alhijawi MM, Lynch E (2017). Combined implant and tooth support: an up-to-date comprehensive overview. Int J Dent.

[12] Mamalis A, Markopoulou K, Kaloumenos K, Analitis A (2012). Splinting osseointegrated implants and natural teeth in partially edentulous patients: a systematic review of the literature. J Oral Implantol.

[13] Eskitascioglu G, Usumez A, Sevimay M, Soykan E, Unsal E (2004). The influence of occlusal loading location on stresses transferred to implant-supported prostheses and supporting bone: a three-dimensional finite element study. J Prosthet Dent.

[14] Menicucci G, Mossolov A, Mozzati M, Lorenzetti M, Preti G (2002). Tooth-implant connection: some biomechanical aspects based on finite element analyses. Clin Oral Implants Res.

[15] Shurbaji Mozayek R, Allaf M, M BA (2016). Efficacy of adding a supporting implant in stress distribution of long-span fixed partial dentures: a 3D finite element analysis. J Dent Res Dent Clin Dent Prospects.

[16] Lindh T (2008). Should we extract teeth to avoid tooth-implant combinations?. J Oral Rehabil.

[17] Guarnieri R, Ippoliti S (2019). Long-term outcomes of tooth-implant-supported rehabilitation of periodontally compromised and treated patients refusing bone grafting surgical therapies. Implant Dent.

[18] Chrcanovic BR, Kisch J, Larsson C (2020). Analysis of technical complications and risk factors for failure of combined tooth-implant-supported fixed dental prostheses. Clin Implant Dent Relat Res.

[19] Ting M, Faulkner RJ, Donatelli DP, Suzuki JB (2019). Tooth-to-implant-supported fixed partial denture: a comprehensive overview of systematic reviews. Implant Dent.

[20] La Monaca G, Pranno N, Annibali S, Massimo C, Polimeni A, Patini R (2020). Survival and complication rates of tooth-implant versus freestanding implant supporting fixed partial prosthesis: a systematic review and meta-analysis. J Prosthodont Res.

[21] Fobbe H, Rammelsberg P, Lorenzo Bermejo J, Kappel S (2019). The up-to-11-year survival and success of implants and abutment teeth under solely implant-supported and combined tooth-implant-supported double crown-retained removable dentures. Clin Oral Implants Res.

[22] Geng JP, Tan KB, Liu GR (2001). Application of finite element analysis in implant dentistry: a review of the literature. J Prosthet Dent.

[23] Gultekin BA, Gultekin P, Yalcin S. Application of finite element analysis in implant dentistry. In: Finite Element Analysis: New Trends and Developments. Rijeka, Croatia: InTech; 2012.

[24] Lekholm U, Zarb GA. Patient selection and preparation. In: Tissue-Integrated Prostheses: Osseointegration in Clinical Dentistry. Chicago: Quintessence Publishing Company; 1985. p. 199-209.

[25] Benzing UR, Gall H, Weber H (1995). Biomechanical aspects of two different implant-prosthetic concepts for edentulous maxillae. Int J Oral Maxillofac Implants.

[26] Holmes DC, Diaz-Arnold AM, Leary JM (1996). Influence of post dimension on stress distribution in dentin. J Prosthet Dent.

[27] Zarone F, Sorrentino R, Apicella D, Valentino B, Ferrari M, Aversa R (2006). Evaluation of the biomechanical behavior of maxillary central incisors restored by means of endocrowns compared to a natural tooth: a 3D static linear finite elements analysis. Dent Mater.

[28] Anusavice KJ. Phillips Materiais Dentários. 12^th^ ed. Elsevier Ltd; 2013. p. 2061.

[29] Ruben JL, Roeters FJM, Truin GJ, Loomans BAC, Huysmans M (2019). Cup-shaped tooth wear defects: more than erosive challenges?. Caries Res.

[30] Pedrollo Lise D, Van Ende A, De Munck J, Umeda Suzuki TY, Cardoso Vieira LC, Van Meerbeek B (2017). Biomechanical behavior of endodontically treated premolars using different preparation designs and CAD/CAM materials. J Dent.

[31] Lin CL, Wang JC, Chang WJ (2008). Biomechanical interactions in tooth-implant-supported fixed partial dentures with variations in the number of splinted teeth and connector type: a finite element analysis. Clin Oral Implants Res.

[32] Lin CL, Wang JC, Chang SH, Chen ST (2010). Evaluation of stress induced by implant type, number of splinted teeth, and variations in periodontal support in tooth-implant-supported fixed partial dentures: a non-linear finite element analysis. J Periodontol.

[33] Lanza MD, Seraidarian PI, Jansen WC, Lanza MD (2011). Stress analysis of a fixed implant-supported denture by the finite element method (FEM) when varying the number of teeth used as abutments. J Appl Oral Sci.

[34] Yang HS, Lang LA, Felton DA (1999). Finite element stress analysis on the effect of splinting in fixed partial dentures. J Prosthet Dent.

[35] Becker CM, Kaiser DA, Jones JD (2000). Guidelines for splinting implants. J Prosthet Dent.

[36] Bates JF, Stafford GD, Harrison A (1976). Masticatory function--a review of the literature III Masticatory performance and efficiency. J Oral Rehabil.

[37] Bates JF, Stafford GD, Harrison A (1975). Masticatory function-a review of the literature: (II) Speed of movement of the mandible, rate of chewing and forces developed in chewing. J Oral Rehabil.

[38] van der Bilt A, Engelen L, Pereira LJ, van der Glas HW, Abbink JH (2006). Oral physiology and mastication. Physiol Behav.

[39] DeLong R, Douglas WH (1991). An artificial oral environment for testing dental materials. IEEE Trans Biomed Eng.

[40] Hidaka O, Morimoto T, Kato T, Masuda Y, Inoue T, Takada K (1999). Behavior of jaw muscle spindle afferents during cortically induced rhythmic jaw movements in the anesthetized rabbit. J Neurophysiol.

[41] Pesun IJ (1997). Intrusion of teeth in the combination implant-to-natural-tooth fixed partial denture: a review of the theories. J Prosthodont.

[42] 42 Nyman SR, Lang NP (1994). Tooth mobility and the biological rationale for splinting teeth. Periodontol 2000.

[43] Naert IE, Duyck JA, Hosny MM, Quirynen M, van Steenberghe D (2001). Freestanding and tooth-implant connected prostheses in the treatment of partially edentulous patients Part II: an up to 15-years radiographic evaluation. Clin Oral Implants Res.

